# Supplementary material for: B and T Cell Bi-Cistronic Multiepitopic Vaccine Induces Broad Immunogenicity and Provides Protection Against SARS-CoV-2
Source: Vaccines (Basel). 2024 Oct 25;12(11):1213. doi: 10.3390/vaccines12111213 (PMC11598604; doi:10.3390/vaccines12111213)
Supplement: Supplementary file 1 [file vaccines-12-01213-s001.zip › vaccines-3231472-supplementary.pdf]

Supplementary Table S1

Predicted population coverage of MHC class I epitopes for CoV2-TMEP

Population / area	MHC class I coverage[a]	average_hit[b]	pc90[c]
American Samoa	99.97%	58.89	36.32
American Samoa Polynesian	99.97%	58.89	36.32
Argentina	98.73%	26.94	11.76
Argentina Amerindian	98.73%	26.94	11.76
Australia	100.0%	69.71	44.45
Australia Australian Aborigines	99.99%	61.52	37.56
Australia Caucasoid	100.0%	84.03	60.99
Austria	100.0%	44.81	30.61
Austria Caucasoid	100.0%	44.81	30.61
Belgium	99.93%	31.33	18.92
Belgium Caucasoid	99.93%	31.33	18.92
Brazil	100.0%	101.02	72.09
Brazil Amerindian	100.0%	57.2	36.96
Brazil Caucasoid	99.95%	39.43	24.37
Brazil Mixed	100.0%	101.7	71.98
Brazil Other	99.89%	29.54	24.51
Bulgaria	100.0%	83.19	54.86
Bulgaria Caucasoid	99.98%	43.28	27.36
Bulgaria Other	100.0%	77.01	48.51
Burkina Faso	96.1%	47.89	16.65
Burkina Faso Black	96.1%	47.89	16.65
Cameroon	99.95%	71.37	42.22
Cameroon Black	99.95%	71.37	42.22
Cape Verde	99.95%	46.41	28.49
Cape Verde Black	99.95%	46.41	28.49
Central Africa	99.91%	69.27	40.06
Central African Republic	97.2%	43.66	19.2

Central African Republic Black	97.2%	43.66	19.2
Central America	10.38%	1.49	0.45
Chile	100.0%	94.28	63.24
Chile Amerindian	100.0%	83.83	57.13
Chile Mixed	99.6%	55.91	27.24
China	100.0%	104.39	72.79
China Oriental	100.0%	104.39	72.79
Colombia	99.97%	36.64	25.86
Colombia Amerindian	99.15%	32.75	27.55
Colombia Black	99.05%	32.64	25.31
Colombia Mestizo	100.0%	38.11	27.49
Croatia	100.0%	44.54	29.54
Croatia Caucasoid	100.0%	44.54	29.54
Cuba	99.94%	41.71	24.93
Cuba Caucasoid	99.96%	41.27	25.09
Cuba Mulatto	99.88%	42.0	24.39
Czech Republic	100.0%	71.98	46.59
Czech Republic Caucasoid	100.0%	71.98	46.59
Denmark	100.0%	31.29	25.12
Denmark Caucasoid	100.0%	31.29	25.12
East Africa	100.0%	96.75	65.3
East Asia	100.0%	151.29	117.88
Ecuador	77.35%	10.22	4.86
Ecuador Amerindian	77.35%	10.22	4.86
England	100.0%	100.74	68.97
England Caucasoid	100.0%	110.19	81.98
England Jew	77.15%	23.19	3.94
Equatorial Guinea	44.5%	14.7	4.5
Equatorial Guinea Black	44.5%	14.7	4.5
Europe	100.0%	147.47	115.85
Finland	100.0%	87.79	65.67

Finland Caucasoid	100.0%	87.79	65.67
France	100.0%	86.41	61.56
France Caucasoid	100.0%	86.41	61.56
Georgia	100.0%	78.14	50.62
Georgia Caucasoid	100.0%	79.69	53.17
Georgia Kurd	100.0%	79.86	51.42
Germany	100.0%	113.64	88.8
Germany Caucasoid	100.0%	113.64	88.8
Ghana	99.77%	24.37	23.46
Ghana Black	99.77%	24.37	23.46
Guatemala	10.38%	1.49	0.45
Guatemala Amerindian	10.38%	1.49	0.45
Guinea-Bissau	99.62%	38.16	21.57
Guinea-Bissau Black	99.62%	38.16	21.57
Hong Kong	99.62%	42.19	19.3
Hong Kong Oriental	99.62%	42.19	19.3
India	100.0%	128.7	91.76
India Asian	100.0%	128.7	91.76
Indonesia	96.91%	28.91	11.57
Indonesia Austronesian	96.91%	28.91	11.57
Iran	100.0%	104.5	72.93
Iran Persian	100.0%	104.5	72.93
Ireland Northern	100.0%	86.02	64.36
Ireland Northern Caucasoid	100.0%	86.02	64.36
Ireland South	100.0%	87.01	64.79
Ireland South Caucasoid	100.0%	87.01	64.79
Israel	99.87%	64.73	34.64
Israel Arab	99.98%	70.65	41.94
Israel Jew	99.96%	69.71	39.59
Italy	100.0%	84.11	58.5
Italy Caucasoid	100.0%	84.11	58.5

Ivory Coast	76.18%	13.37	2.94
Ivory Coast Black	76.18%	13.37	2.94
Japan	100.0%	148.01	114.91
Japan Oriental	100.0%	148.01	114.91
Jordan	98.68%	48.53	19.65
Jordan Arab	98.68%	48.53	19.65
Kenya	99.97%	74.73	47.34
Kenya Black	99.97%	74.73	47.34
Korea; South	100.0%	152.08	119.69
Korea; South Oriental	100.0%	152.08	119.69
Lebanon	99.76%	40.86	20.1
Lebanon Mixed	99.76%	40.86	20.1
Macedonia	71.29%	23.42	1.39
Macedonia Caucasoid	71.29%	23.42	1.39
Malaysia	96.91%	30.49	12.45
Malaysia Austronesian	89.96%	25.07	3.99
Malaysia Oriental	98.61%	33.76	15.19
Mali	100.0%	81.65	55.36
Mali Black	100.0%	81.65	55.36
Martinique	22.56%	2.71	1.55
Martinique Black	22.56%	2.71	1.55
Mexico	100.0%	107.16	77.59
Mexico Amerindian	100.0%	108.27	79.49
Mexico Mestizo	100.0%	73.44	49.7
Mongolia	95.48%	18.56	11.2
Mongolia Oriental	95.48%	18.56	11.2
Morocco	100.0%	78.31	52.56
Morocco Arab	100.0%	76.43	50.37
Morocco Caucasoid	100.0%	79.64	54.2
New Caledonia	99.99%	59.26	36.68
New Caledonia Melanesian	99.99%	59.26	36.68

North Africa	99.99%	77.64	49.71
North America	100.0%	143.26	110.63
Northeast Asia	100.0%	105.37	73.77
Oceania	99.94%	61.08	35.37
Oman	99.93%	43.85	25.98
Oman Arab	99.93%	43.85	25.98
Pakistan	99.9%	56.28	26.02
Pakistan Asian	99.9%	55.81	26.03
Pakistan Mixed	99.89%	56.91	26.0
Papua New Guinea	99.99%	63.63	39.3
Papua New Guinea Melanesian	99.99%	63.63	39.3
Peru	100.0%	35.05	15.6
Peru Amerindian	100.0%	31.12	15.44
Peru Mestizo	15.54%	3.92	2.96
Philippines	100.0%	77.85	53.34
Philippines Austronesian	100.0%	77.85	53.34
Poland	100.0%	114.69	89.98
Poland Caucasoid	100.0%	114.69	89.98
Portugal	99.99%	69.62	41.47
Portugal Caucasoid	99.99%	69.62	41.47
Romania	99.99%	43.8	28.24
Romania Caucasoid	99.99%	43.8	28.24
Russia	100.0%	81.84	56.65
Russia Caucasoid	95.72%	40.79	24.76
Russia Mixed	89.28%	33.61	3.73
Russia Other	100.0%	87.82	63.23
Russia Siberian	100.0%	85.07	61.06
Rwanda	63.36%	11.76	1.09
Rwanda Black	63.36%	11.76	1.09
Sao Tome and Principe	99.17%	35.24	18.11
Sao Tome and Principe Black	99.17%	35.24	18.11

Saudi Arabia	99.99%	73.81	48.68
Saudi Arabia Arab	99.99%	73.81	48.68
Scotland	81.93%	23.4	2.21
Scotland Caucasoid	81.93%	23.4	2.21
Senegal	99.98%	76.67	46.78
Senegal Black	99.98%	76.67	46.78
Serbia	99.44%	21.94	10.67
Serbia Caucasoid	99.44%	21.94	10.67
Singapore	99.93%	72.7	42.74
Singapore Austronesian	99.9%	67.92	39.54
Singapore Oriental	99.99%	82.03	53.44
South Africa	99.97%	78.98	50.42
South Africa Black	97.48%	33.18	13.13
South Africa Other	99.99%	80.83	54.17
South America	100.0%	121.7	87.08
South Asia	100.0%	133.07	96.15
Southeast Asia	100.0%	114.36	83.48
Southwest Asia	99.99%	87.48	54.71
Spain	100.0%	134.12	103.04
Spain Caucasoid	100.0%	134.12	103.04
Sri Lanka	49.02%	6.93	2.16
Sri Lanka Asian	49.02%	6.93	2.16
Sudan	99.99%	75.38	47.95
Sudan Arab	90.64%	29.72	6.71
Sudan Black	14.75%	1.21	0.47
Sudan Mixed	99.87%	67.56	36.79
Sweden	100.0%	41.14	26.96
Sweden Caucasoid	100.0%	41.14	26.96
Switzerland	98.23%	39.89	20.68
Switzerland Caucasoid	98.23%	39.89	20.68
Taiwan	100.0%	82.11	55.64

Taiwan Oriental	100.0%	82.11	55.64
Thailand	100.0%	115.0	84.53
Thailand Oriental	100.0%	115.0	84.53
Trinidad and Tobago	0.0%	0.0	3.2
Trinidad and Tobago Asian	0.0%	0.0	3.2
Tunisia	99.99%	79.63	51.72
Tunisia Arab	99.99%	79.63	51.72
Turkey	80.65%	28.8	4.65
Turkey Caucasoid	80.65%	28.8	4.65
Uganda	99.98%	75.31	47.06
Uganda Black	99.98%	75.31	47.06
United Arab Emirates	14.44%	1.68	0.47
United Arab Emirates Arab	14.44%	1.68	0.47
United Kingdom	99.99%	43.43	27.14
United Kingdom Caucasoid	99.99%	43.43	27.14
United States	100.0%	143.66	111.09
United States Amerindian	100.0%	74.94	47.97
United States Asian	100.0%	83.87	57.61
United States Black	100.0%	139.02	105.73
United States Caucasoid	100.0%	112.96	87.57
United States Hispanic	100.0%	81.0	55.38
United States Mestizo	100.0%	81.99	56.77
United States Polynesian	100.0%	84.84	60.67
Venezuela	99.94%	60.3	38.38
Venezuela Amerindian	99.94%	59.54	38.27
Venezuela Caucasoid	15.83%	2.47	1.31
Venezuela Mestizo	13.4%	2.23	1.39
Vietnam	99.98%	79.85	51.15
Vietnam Oriental	99.98%	79.85	51.15
Wales	6.88%	0.48	0.43
Wales Caucasoid	6.88%	0.48	0.43

West Africa	100.0%	106.49	78.02
West Indies	99.94%	41.74	25.05
World	100.0%	147.5	113.81
Zambia	100.0%	99.79	71.71
Zambia Black	100.0%	99.79	71.71
Zimbabwe	100.0%	106.54	77.06
Zimbabwe Black	100.0%	106.54	77.06
**Average**	**91.16**	**62.99**	**41.47**
**Standard deviation**	**23.37**	**37.44**	**30.06**

[a] projected population coverage; [b] average number of epitope hits / HLA combinations recognized by the population; [c] minimum number of epitope hits / HLA combinations recognized by 90% of the population.

Supplementary Table S2

Predicted population coverage of MHC class II epitopes for CoV2-TMEP

	MHC class II		
**Population / Area**	**coverage[a]**	**average_hit[b]**	**pc90[c]**
Algeria	98.8%	17.47	9.35
Algeria Arab	98.8%	17.47	9.35
Argentina	99.46%	18.4	10.49
Argentina Amerindian	99.96%	18.59	10.73
Argentina Caucasoid	97.6%	17.05	7.98
Australia	99.99%	18.44	10.76
Australia Australian Aborigines	99.99%	18.44	10.76
Austria	98.1%	17.76	7.99
Austria Caucasoid	98.1%	17.76	7.99
Belarus	45.3%	4.02	1.28
Belarus Caucasoid	45.3%	4.02	1.28
Belgium	92.49%	14.19	7.03
Belgium Caucasoid	92.49%	14.19	7.03
Bolivia	99.98%	17.1	9.79
Bolivia Amerindian	99.98%	17.1	9.79
Borneo	99.94%	18.09	9.56
Borneo Austronesian	99.94%	18.09	9.56
Brazil	99.79%	18.78	11.73
Brazil Amerindian	100.0%	18.7	12.16
Brazil Caucasoid	98.75%	18.44	11.01
Brazil Mixed	98.81%	17.87	9.25
Brazil Mulatto	97.01%	17.17	9.06
Bulgaria	98.39%	19.05	9.67
Bulgaria Caucasoid	98.39%	19.05	9.67
Cameroon	95.38%	15.52	7.64
Cameroon Black	95.38%	15.52	7.64
Canada	97.95%	14.51	9.08

Canada Amerindian	97.95%	14.51	9.08
Cape Verde	95.23%	15.53	7.35
Cape Verde Black	95.23%	15.53	7.35
Central Africa	94.62%	15.79	7.41
Central African Republic	94.98%	14.38	7.32
Central African Republic Black	94.98%	14.38	7.32
Central America	99.83%	16.97	9.5
Chile	96.24%	15.24	7.85
Chile Amerindian	97.28%	13.36	8.14
Chile Mixed	85.79%	12.28	4.22
China	99.67%	18.63	11.41
China Oriental	99.67%	18.63	11.41
Colombia	99.5%	18.03	10.23
Colombia Amerindian	99.83%	18.02	10.62
Colombia Black	98.28%	17.04	8.02
Colombia Mestizo	91.59%	14.6	6.55
Congo	82.6%	11.76	2.87
Congo Black	82.6%	11.76	2.87
Cook Islands	99.97%	16.82	9.56
Cook Islands Polynesian	99.97%	16.82	9.56
Costa Rica	99.84%	17.51	9.89
Costa Rica Mestizo	99.84%	17.51	9.89
Croatia	96.48%	18.09	8.78
Croatia Caucasoid	96.48%	18.09	8.78
Cuba	98.23%	17.71	11.04
Cuba Mixed	98.23%	17.71	11.04
Czech Republic	98.26%	17.79	9.41
Czech Republic Caucasoid	98.32%	18.03	9.49
Czech Republic Other	97.16%	15.36	9.03
Denmark	91.71%	14.1	7.11
Denmark Caucasoid	91.71%	14.1	7.11

East Africa	97.34%	16.55	8.98
East Asia	99.43%	18.44	10.85
Ecuador	100.0%	18.36	10.18
Ecuador Amerindian	100.0%	18.36	10.18
England	99.2%	17.56	7.99
England Caucasoid	99.2%	17.56	7.99
Equatorial Guinea	82.61%	12.59	2.88
Equatorial Guinea Black	82.61%	12.59	2.88
Ethiopia	95.12%	15.5	7.68
Ethiopia Black	95.12%	15.5	7.68
Europe	98.8%	18.21	9.55
Fiji	99.99%	17.52	9.71
Fiji Melanesian	99.99%	17.52	9.71
Finland	62.05%	7.71	1.58
Finland Caucasoid	62.05%	7.71	1.58
France	98.38%	17.31	7.78
France Caucasoid	98.38%	17.31	7.78
Gabon	55.91%	6.82	2.04
Gabon Black	55.91%	6.82	2.04
Georgia	97.55%	16.75	9.07
Georgia Caucasoid	97.55%	16.75	9.07
Germany	98.29%	17.69	7.95
Germany Caucasoid	98.29%	17.69	7.95
Greece	99.33%	20.02	11.65
Greece Caucasoid	99.33%	20.02	11.65
Guatemala	98.31%	14.36	9.16
Guatemala Amerindian	98.31%	14.36	9.16
Guinea-Bissau	90.76%	14.17	7.15
Guinea-Bissau Black	90.76%	14.17	7.15
India	98.94%	18.32	10.99
India Asian	98.94%	18.32	10.99

Indonesia	99.96%	17.93	9.7
Indonesia Austronesian	99.96%	17.93	9.7
Iran	99.05%	16.77	7.87
Iran Kurd	99.19%	16.26	7.79
Iran Persian	98.96%	16.75	7.86
Ireland Northern	99.34%	17.72	7.98
Ireland Northern Caucasoid	99.34%	17.72	7.98
Ireland South	99.06%	16.92	7.74
Ireland South Caucasoid	99.06%	16.92	7.74
Israel	97.84%	16.9	8.53
Israel Arab	98.36%	16.93	8.78
Israel Jew	97.61%	16.85	8.24
Italy	99.33%	15.31	7.44
Italy Caucasoid	99.33%	15.31	7.44
Jamaica	76.28%	9.38	2.11
Jamaica Black	76.28%	9.38	2.11
Japan	99.65%	18.99	11.26
Japan Oriental	99.65%	18.99	11.26
Jordan	99.48%	13.58	6.53
Jordan Arab	99.48%	13.58	6.53
Kiribati	100.0%	16.92	9.41
Kiribati Micronesian	100.0%	16.92	9.41
Korea; South	98.84%	17.72	9.85
Korea; South Oriental	98.84%	17.72	9.85
Lebanon	99.3%	18.21	9.67
Lebanon Arab	99.3%	18.21	9.67
Macedonia	99.04%	19.89	11.46
Macedonia Caucasoid	99.04%	19.89	11.46
Malaysia	99.87%	19.08	11.62
Malaysia Austronesian	99.95%	18.82	11.46
Malaysia Oriental	99.45%	18.59	11.37

Martinique	97.28%	16.92	7.94
Martinique Black	97.28%	16.92	7.94
Mexico	99.85%	18.31	10.6
Mexico Amerindian	99.97%	18.55	10.87
Mexico Mestizo	99.67%	18.04	9.95
Mongolia	99.71%	18.47	11.51
Mongolia Oriental	99.71%	18.47	11.51
Morocco	97.23%	15.69	7.63
Morocco Arab	96.59%	15.19	7.5
Morocco Caucasoid	98.04%	16.52	7.97
Nauru	99.98%	17.4	9.84
Nauru Micronesian	99.98%	17.4	9.84
Netherlands	96.41%	16.12	7.44
Netherlands Caucasoid	96.41%	16.12	7.44
New Caledonia	100.0%	18.21	9.81
New Caledonia Melanesian	100.0%	18.21	9.81
New Zealand	99.91%	16.98	9.56
New Zealand Polynesian	99.91%	16.98	9.56
Niue	99.98%	15.84	8.02
Niue Polynesian	99.98%	15.84	8.02
North Africa	98.08%	16.88	8.0
North America	98.7%	17.71	9.07
Northeast Asia	99.67%	18.63	11.41
Norway	98.31%	16.77	7.6
Norway Caucasoid	98.31%	16.77	7.6
Oceania	99.98%	19.28	15.75
Pakistan	7.05%	0.92	1.4
Pakistan Asian	6.68%	0.87	1.39
Pakistan Mixed	7.84%	1.02	1.41
Papua New Guinea	100.0%	19.63	15.26
Papua New Guinea Melanesian	100.0%	19.63	15.26

Paraguay	100.0%	16.56	10.57
Paraguay Amerindian	100.0%	16.56	10.57
Peru	99.6%	17.3	9.7
Peru Amerindian	99.6%	17.3	9.7
Philippines	100.0%	17.52	11.15
Philippines Austronesian	100.0%	17.52	11.15
Poland	98.4%	18.46	9.38
Poland Caucasoid	98.4%	18.46	9.38
Portugal	97.82%	17.22	8.18
Portugal Caucasoid	97.82%	17.22	8.18
Russia	99.48%	18.68	11.09
Russia Caucasoid	98.92%	18.7	10.22
Russia Other	99.83%	19.68	11.82
Russia Siberian	99.49%	18.2	10.75
Rwanda	93.08%	14.99	7.09
Rwanda Black	93.08%	14.99	7.09
Samoa	99.93%	17.57	11.14
Samoa Polynesian	99.93%	17.57	11.14
Sao Tome and Principe	97.62%	16.63	7.59
Sao Tome and Principe Black	97.62%	16.63	7.59
Saudi Arabia	96.58%	15.73	7.84
Saudi Arabia Arab	96.58%	15.73	7.84
Scotland	96.39%	15.49	7.39
Scotland Caucasoid	96.39%	15.49	7.39
Senegal	81.94%	12.59	2.77
Senegal Black	81.94%	12.59	2.77
Singapore	99.9%	17.92	9.67
Singapore Austronesian	99.9%	17.92	9.67
Slovakia	22.38%	1.87	0.9
Slovakia Caucasoid	22.38%	1.87	0.9
Slovenia	98.65%	18.76	9.65

Slovenia Caucasoid	98.65%	18.76	9.65
South Africa	53.35%	3.84	1.07
South Africa Black	53.35%	3.84	1.07
South America	99.74%	18.67	11.1
South Asia	99.01%	18.35	11.02
Southeast Asia	99.9%	18.8	11.94
Southwest Asia	99.15%	15.18	6.83
Spain	98.45%	17.69	9.44
Spain Caucasoid	98.45%	17.69	9.43
Spain Jew	0.0%	0.0	1.4
Spain Other	25.61%	2.95	1.48
Sudan	95.16%	16.08	7.52
Sudan Mixed	95.16%	16.08	7.52
Sweden	98.83%	18.82	9.7
Sweden Caucasoid	98.83%	18.82	9.7
Taiwan	99.98%	18.51	13.06
Taiwan Oriental	99.98%	18.51	13.06
Thailand	99.88%	19.01	11.65
Thailand Oriental	99.88%	19.01	11.65
Tokelau	99.91%	14.95	8.22
Tokelau Polynesian	99.91%	14.95	8.22
Tonga	100.0%	18.15	11.51
Tonga Polynesian	100.0%	18.15	11.51
Tunisia	98.07%	16.54	7.92
Tunisia Arab	98.07%	16.15	7.82
Tunisia Berber	97.08%	17.23	8.87
Turkey	98.46%	17.75	9.29
Turkey Caucasoid	98.46%	17.75	9.29
Ukraine	50.64%	4.51	1.42
Ukraine Caucasoid	50.64%	4.51	1.42
United Arab Emirates	34.71%	2.41	0.77

United Arab Emirates Arab	34.71%	2.41	0.77
United States	98.61%	17.65	8.23
United States Amerindian	99.99%	17.6	10.59
United States Asian	99.61%	18.54	11.29
United States Austronesian	99.96%	17.95	11.31
United States Black	96.91%	16.54	7.73
United States Caucasoid	98.08%	17.15	7.75
United States Hispanic	98.99%	17.63	9.7
United States Mestizo	99.29%	17.96	9.94
United States Polynesian	99.95%	18.33	11.82
Venezuela	100.0%	16.87	10.51
Venezuela Amerindian	100.0%	16.4	10.46
Venezuela Mixed	10.13%	1.14	0.78
Vietnam	99.85%	18.14	9.9
Vietnam Oriental	99.85%	18.14	9.9
Wales	4.35%	0.61	1.46
Wales Caucasoid	4.35%	0.61	1.46
West Africa	92.93%	15.23	7.13
West Indies	98.29%	17.32	9.3
World	99.09%	18.24	10.07
Zimbabwe	97.34%	16.55	8.98
Zimbabwe Black	97.34%	16.55	8.98
**Average**	**91.59**	**15.7**	**8.38**
**Standard deviation**	**20.44**	**4.43**	**3.02**

[a] projected population coverage; [b] average number of epitope hits / HLA combinations recognized by the population; [c] minimum number of epitope hits / HLA combinations recognized by 90% of the population.
